# Klymollins T–X, Bioactive Eunicellin-Based Diterpenoids from the Soft Coral *Klyxum molle*

**DOI:** 10.3390/md12053060

**Published:** 2014-05-22

**Authors:** Fang-Yu Chang, Fang-Jung Hsu, Chi-Jen Tai, Wen-Chi Wei, Ning-Sun Yang, Jyh-Horng Sheu

**Affiliations:** 1 Department of Marine Biotechnology and Resources, National Sun Yat-sen University, Kaohsiung 804, Taiwan; E-Mails: tako0225j@gmail.com (F.-Y.C.); fangjungh@gmail.com (F.-J.H.); jean801023@hotmail.com (C.-J.T.); 2 Agricultural Biotechnology Research Center, Academia Sinica, 128 Academia Road, Section 2, Nankang, Taipei 115, Taiwan; E-Mail: jackwei@gate.sinica.edu.tw; 3 Institute of Biotechnology, National Taiwan University, Taipei 106, Taiwan; 4 Department of Life Science, National Central University, Taoyuan 320, Taiwan; 5 Division of Marine Biotechnology, Asia-Pacific Ocean Research Center, National Sun Yat-sen University, Kaohsiung 804, Taiwan; 6 Doctoral Degree Program in Marine Biotechnology, National Sun Yat-sen University and Academia Sinica, Kaohsiung 804, Taiwan; 7 Department of Medical Research, China Medical University Hospital, China Medical University, Taichung 404, Taiwan; 8 Graduate Institute of Natural Products, Kaohsiung Medical University, Kaohsiung 807, Taiwan

**Keywords:** soft coral, *Klyxum molle*, eunicellin-based diterpenoids, klymollins, cytotoxic activity, anti-inflammatory activity

## Abstract

Five new eunicellin-based diterpenoids, klymollins T–X (**1**–**5**), along with two known compounds (**6** and **7**) have been isolated from the soft coral *Klyxum molle*. The structures of these new metabolites were elucidated by extensive spectroscopic analysis and by comparison with related known compounds. Compound **5** was found to exert significant *in vitro* anti-inflammatory activity against LPS-stimulated RAW264.7 macrophage cells. Furthermore, compounds **4** and **7** were shown to exhibit cytotoxicity against a limited panel of human cancer cell lines.

## 1. Introduction

**Chart 1:**
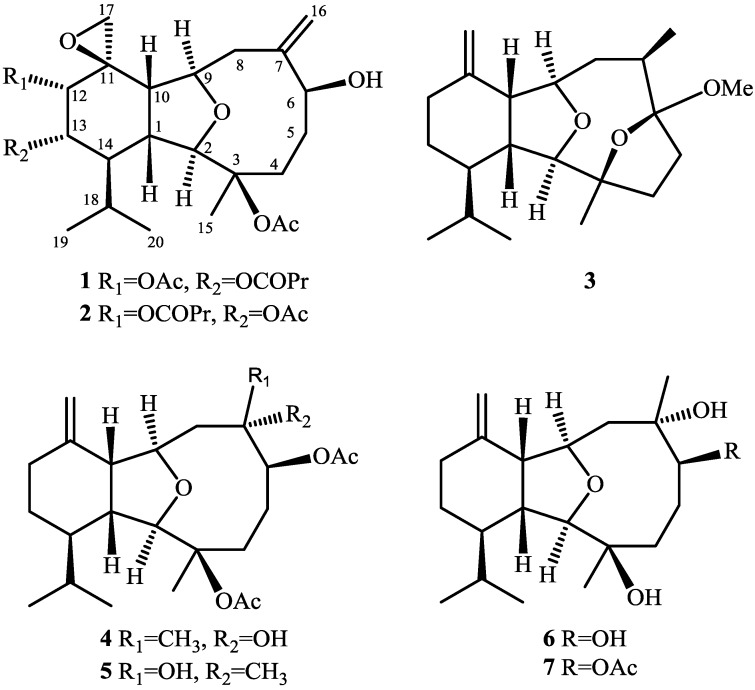
Structures of metabolites **1**–**7**.

Soft corals are known to be a rich source of terpenoidal metabolites [1]. Many studies about the discovery of versatile molecular structures and bioactivities of eunicellin-type compounds from soft corals have been reported recently [2,3,4,5,6,7,8,9,10,11,12,13,14,15,16,17,18]. Our previous studies on the secondary metabolites of a Formosan soft coral *Klyxum molle* have resulted in the isolation of a series of new eunicellin-based diterpenoids, klymollins A–S [19,20]. In our continuing investigation effort to discover new metabolites from the soft coral *K. molle*, we have identified five new eunicellin-type metabolites, klymollins T–X (**1**–**5**) (Chart 1 and Supplementary Figures S1–S15), along with two known eunicellin-based diterpenoids, sclerophytin A (**6**) and sclerophytin B (**7**) [21] (Chart 1). The molecular structures of these compounds, including their relative configurations, were established by detailed spectroscopic analysis and by comparison with related physical and spectral data of known compounds. The ability of compounds **1**–**7** to inhibit IL-6 (interlukin-6) and TNF-α (tumor necrosis factor α) expression in LPS (lipopolysaccharide)-stimulated murine RAW264.7 macrophage cells and the cytotoxicity of **3**–**7** against five human cancer cell lines, human T cell lymphoblast-like cell line (CCRF-CEM), human erythromyeloblastoid leukemia (K562), human acute lymphoblastic leukemia cell line (Molt 4), human ductal breast epithelial tumor cell line (T47D) and human colorectal adenocarcinoma cell line (DLD-1) were evaluated.

## 2. Results and Discussion

**Figure 1:**
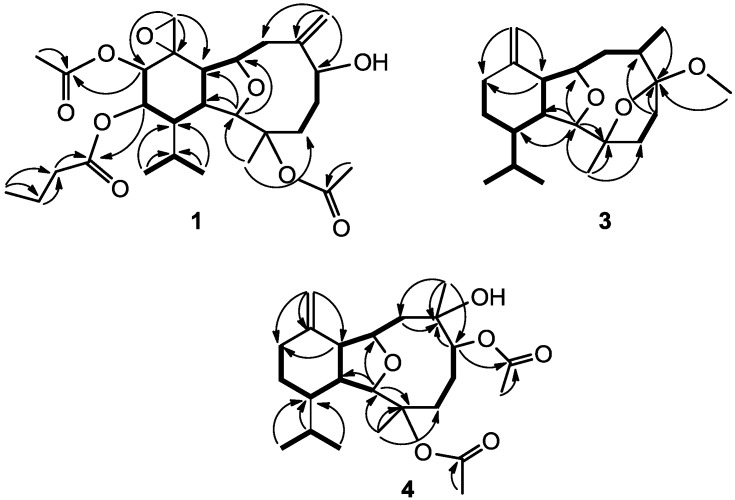
Selected COSY (▬) and HMBC (→) correlations of **1**, **3** and **4**.

Klymollin T (**1**) was obtained as a colorless oil. The HRESIMS (*m*/*z* 545.2722 [M + Na]^+^) of **1** provided its molecular formula as C_28_H_42_O_9_, implying the presence of eight degrees of unsaturation. The IR spectrum of **1** revealed the presence of hydroxy and carbonyl groups from absorptions at 3481 and 1746 cm^–1^, respectively. The ^13^C NMR spectroscopic data of **1** exhibited 28 carbon signals (Table 1), which were assigned by the aid of DEPT spectrum to six methyls (including two acetate methyl at δ_C_ 21.2 and 20.8), seven methylenes (including one oxymethylene at δ_C_ 53.0), nine methines (including five oxymethines at δ_C_ 91.6, 80.6, 74.2, 73.0 and 72.9), and six quaternary carbons (including three sp^2^ oxygenated quaternary carbons at δ_C_ 172.0, 170.1 and 170.1, two sp^3^ oxygenated quaternary carbons at δ_C_ 84.9 and 55.7, and one sp^2^ quaternary carbon at δ_C_ 150.5). The NMR data of **1** in C_6_D_6_ (Table 1) showed the presence of one *n*-butyrate (δ_C_ 172.0, C; 37.8, CH_2_; 19.2, CH_2_; and 14.1, CH_3_; and δ_H_ 1.98, 2H, m; 1.53, 2H, m; and 0.83, 3H, t, *J* = 7.5 Hz), one 1,1-disubstituted double bond (δ_C_ 116.9, CH_2_ and 150.5, C; and δ_H_ 5.24, 1H, d, *J* = 2.0 Hz and 4.86, 1H, brs), one terminal epoxide (δ_C_ 53.0, CH_2_ and 55.7, C; δ_H_ 2.31, d and 1.99, d, each 1H, *J* = 5.0 Hz) and two acetate groups (δ_C_ 170.1, C; 170.1, C; 20.8, CH_3_ and 21.2, CH_3_; and δ_H_ 1.62, s, and 1.82, s, each 3H), respectively. Analysis of HMQC correlations showed that proton signals appearing at δ_H_ 2.33 (1H, m), 2.17 (1H, t, *J* = 8.5 Hz), 3.97 (1H, br s), and 5.00 (1H, d, *J* = 5.5 Hz) were correlated to two ring juncture methine carbons at δ_C_ 42.4 and 43.5 and two oxymethine carbons at δ_C_ 91.6 and 80.6, respectively. Therefore, the remaining three degrees of unsaturation identified **1** as a tricyclic diterpenoid. In addition, the COSY correlations of **1** assigned three isolated consecutive proton spin systems (Figure 1). The molecular framework of **1** was further established by HMBC correlations (Figure 1). Furthermore, H-12 (δ 5.24) and an acetate methyl (δ 1.62) exhibited HMBC correlations to the acetate carbonyl carbon (δ 170.1), and H-13 exhibited HMBC correlation to the *n*-butyrate carbonyl carbon (δ 172.0), revealing the location of an acetate at C-12 and an *n*-butyrate at C-13. The location of an acetate group at C-3 was then deduced by the chemical shifts of C-3 (δ 84.9) and H_3_-15 (δ 1.75). From the above results, the structure of compound **1** was shown to be related to that of the known compound, klymollin C [19]. Comparison of the NMR data of them revealed that the replacement of the acetoxy group at C-13 in klymollin C by an *n*-butyryloxy group in **1**.

**Figure 2:**
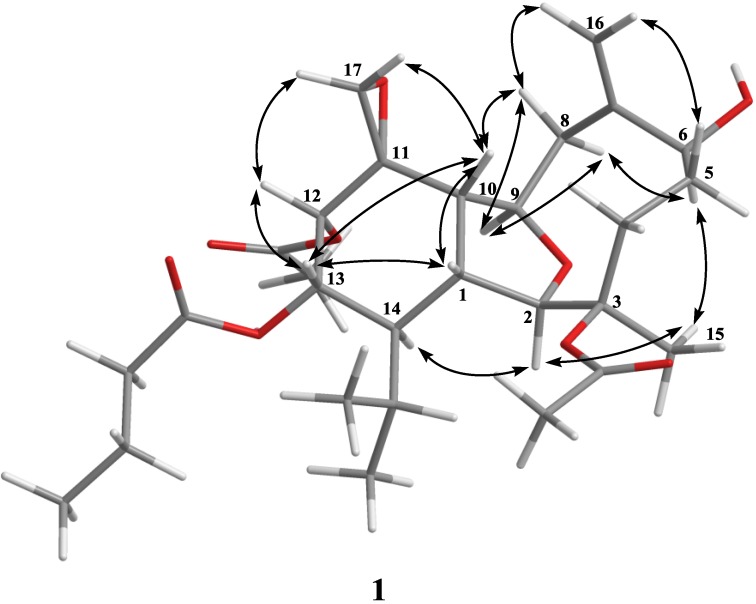
Key NOESY Correlations for **1**.

The relative configuration of **1** was determined by comparison of the chemical shifts with klymollin C and was further confirmed by NOE correlations (Figure 2). The NOE correlations between H-12 and H-13, and between H-13 with H-1, H-10 and H-12 suggested that H-12 and H-13 were β-oriented and the relative configuration of **1** was proposed as 1R*, 2R*, 3R*, 6S*, 9R*, 10S*, 11S*, 12S*, 13S* and 14R*.

The HRESIMS of klymollin U (**2**) exhibited a [M + Na]^+^ peak at *m*/*z* 545.2723 established the same molecular formula as that of **1**. The ^1^H and ^13^C NMR data of **2** (Table 1) were similar to those of **1**, revealed the presence of two acetoxy groups (δ_C_ 170.0, C; 169.4, C; 22.5, CH_3_ and 21.1, CH_3_; and δ_H_ 2.00, s, 3H and 1.92, s, 3H), one *n*-butyryloxy group (δ_C_ 172.6, C; 36.5, CH_2_; 18.8, CH_2_; 13.5, CH_3_; and δ_H_ 2.38, m, 2H; 1.70, m, 2H; 1.00, t, 3H, *J* = 7.6 Hz), one 1,1-disubstituted double bond (δ_C_ 116.8, CH_2_ and 150.0, C; and δ_H_ 5.36, brs and 4.98, brs, each 1H), one terminal epoxide (δ_C_ 53.6, CH_2_ and 55.2, C; δ_H_ 2.87, d and 2.64, d, each 1H, *J* = 4.8 Hz). The positions of one *n*-butyrate group and one acetate group at C-12 and C-13, respectively, was confirmed by the HMBC correlations of H-12 and the oxymethylene protons (δ_H_ 2.38) to the *n*-butyryloxy carbonyl carbon (δ 172.6), and H-13 and an acetate methyl (δ_H_ 2.00) to the acetate carbonyl carbon (δ 170.0). Thus, the remaining one acetate group had to be positioned at C-3, an oxygen-bearing quaternary carbon resonating at δ 84.6 ppm. A more detailed analysis of the ^1^H and ^13^C NMR spectroscopic data and correlations in the ^1^H–^1^H COSY and HMBC spectra led to the establishment of the gross structure of **2** (Figure 1). The stereochemistry of **2** was finally confirmed by comparison of its NMR data and NOE correlations with **1**.

**Table 1:** ^13^C and ^1^H NMR data for compounds **1**–**3**.

	1 ^a^		2 ^b^		3 ^b^	
	δ_H_	δ_C_	δ_H_	δ_C_	δ_H_	δ_C_
1	2.33 m	42.4 (CH) ^c^	2.38 t (7.2) ^d^	41.8 (CH)	2.20 m	46.0 (CH)
2	3.97 brs ^e^	91.6 (CH)	3.79 brs	90.8 (CH)	3.58 brs	90.9 (CH)
3		84.9 (C)		84.6 (C)		86.7 (C)
4	1.49 m	28.5 (CH_2_)	1.53 m	27.8 (CH_2_)	2.10 m	37.6 (CH_2_)
	2.35 m		2.26 m		1.80 t (10.8)	
5	1.62 m	34.4 (CH_2_)	1.69 m	34.3 (CH_2_)	2.76 m	32.8 (CH_2_)
	2.30 m		2.20 m		1.92 t (12.0)	
6	4.24 dd (9.5, 4.0) ^d^	73.0 (CH)	4.29 d (8.0)	72.5 (CH)		113.4 (C)
7		150.5 (C)		150.0 (C)	2.16 m	38.8 (CH)
8	2.57 d (13.5)	41.4 (CH_2_)	2.48 d (13.6)	40.7 (CH_2_)	2.41 ddd (16.0, 6.8, 3.2)	36.0 (CH_2_)
	2.71 dd (13.5, 4.0)		2.78 dd (13.6, 4.0)		1.68 m	
9	5.00 d (5.5)	80.6 (CH)	4.90 m	80.1 (CH)	4.05 dt (8.8, 3.2)	82.1 (CH)
10	2.17 t (8.5)	43.5 (CH)	2.26 t (10.4)	42.9 (CH)	3.92 t (8.0)	46.6 (CH)
11		55.7 (C)		55.2 (C)		148.1 (C)
12	5.24 d (2.0)	74.2 (CH)	4.92 brs	73.6 (CH)	2.02 m	31.2 (CH_2_)
					2.20 m	
13	5.11 dd (11.0, 2.0)	72.9 (CH)	4.91 d (10.4)	72.2 (CH)	0.99 m1.70 m	24.9 (CH_2_)
14	2.11 m	41.8 (CH)	2.01 m	40.7 (CH)	1.26 m	43.2 (CH)
15	1.75 s	22.9 (CH_3_)	1.65 s	22.1 (CH_3_)	1.31 s	23.2 (CH_3_)
16	5.24 d (2.0)	116.9 (CH_2_)	4.98 brs	116.8 (CH_2_)	1.36 d (7.6)	19.1 (CH_3_)
	4.86 brs		5.36 brs			
17	1.99 d (5.0)	53.0 (CH_2_)	2.64 d (4.8)	53.6 (CH_2_)	4.77 brs	109.8 (CH_2_)
	2.31 d (5.0)		2.87 d (4.8)		4.70 d (1.2)	
18	2.06 m	28.3 (CH)	2.03 m	27.3 (CH)	1.70 m	29.1 (CH)
19	0.89 d (7.0)	16.3 (CH_3_)	0.84 d (7.2)	15.3 (CH_3_)	0.95 d (6.8)	21.9 (CH_3_)
20	1.12 d (7.0)	24.5 (CH_3_)	1.09 d (7.2)	24.0 (CH_3_)	0.76 d (6.8)	15.4 (CH_3_)
3-OAc		170.1 (C)		169.4 (C)		
	1.82 s	21.2 (CH_3_)	1.92 s	22.5 (CH_3_)		
6-OMe					3.26 brs	48.5 (CH_3_)
12-OAc		170.1 (C)				
	1.62 s	20.8 (CH_3_)				
12-OCOPr				172.6 (C)		
			2.38 m	36.5 (CH_2_)		
			1.70 m	18.8 (CH_2_)		
			1.00 t (7.6)	13.5 (CH_3_)		
13-OAc				170.0 (C)		
			2.00 s	21.1 (CH_3_)		
13-OCOPr		172.0 (C)				
	1.98 m	37.8 (CH_2_)				
	1.53 m	19.2 (CH_2_)				
	0.83 t (7.5)	14.1 (CH_3_)				

^a^ ^13^C and ^1^H spectra recorded at 125 and 500 MHz in C_6_D_6_; ^b^ ^13^C and ^1^H spectra recorded at 100 and 400 MHz in CDCl_3_; ^c^ Deduced from DEPT; ^d^ *J* values (Hz) in parentheses; ^e^ Broad signal.

**Figure 3:**
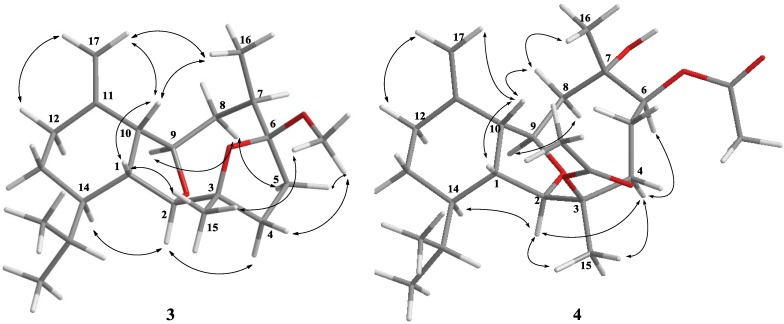
Key NOESY Correlations for **3** and **4**.

Molecular formula C_21_H_34_O_3_ with five degrees of unsaturation was assigned to klymollin V (**3**) from its HRESIMS data (*m*/*z* 357.2405 [M + Na]^+^). The NMR spectroscopic data of **3** (Table 1) showed the presence of one 1,1-disubstituted double bond (δ_C_ 109.8, CH_2_ and 148.1, C; δ_H_ 4.77, brs and 4.70, d, *J* = 1.2 Hz, each 1H) and a methoxyl group (δ_H_ 3.26, 3H, brs). Analysis of HMQC, COSY and HMBC correlations (Figure 1) showed that proton signals appearing at δ_H_ 2.20 (1H, m), 3.92 (1H, t, *J* = 8.0 Hz), 3.58 (1H, brs), and 4.05 (1H, dt, *J* = 8.8 and 3.2 Hz) were correlated to two ring-juncture methine carbons at δ_C_ 46.0 and 46.6 and two oxymethine carbons at δ_C_ 90.9 and 82.1, respectively. Furthermore, one oxygenated quaternary carbon δ_C_ 86.7 (C-3) and one deoxygenated quaternary carbon δ_C_ 113.4 (C-6), implied that C-3 and C-6 were linked through an oxygen to form a tetrahydrofuran ring. The HMBC correlation of the methoxyl protons (δ 3.26) to C-6 (δ 113.4) suggested the substitution of a methoxyl group at C-6. Thus, the molecular framework of **3** was established. The relative stereochemistry of **3** was deduced by careful interpretation of the NOE correlations (Figure 3). The key NOE correlations of **3** showed interactions between H-1 and H-10, and H_3_-15; H-10 and H_3_-16 and H-17a (δ 4.77); and H_3_-15 and 6-OMe. Thus, all of H-1, H-10, H_3_-15, H_3_-16 and 6-OMe should be the β face. NOE correlations were also detected between H-14 and H-2, H-2 and H-4α (δ 2.10), revealing the α-orientation of both H-2 and H-14, as suggested by a molecular model of **3** (Figure 3). On the basis of the above findings, the structure of compound **3**, including the relative stereochemistry, was unambiguously established.

Klymollin W (**4**) showed the pseudomolecular ion peak [M + Na]^+^ at *m*/*z* 445.2563 in the HRESIMS and the molecular formula was determined as C_24_H_38_O_6_. NMR spectroscopic data of **4** (Table 2) showed the presence of two acetoxy groups (δ_C_ 171.9, 169.6, 22.4 and 21.4; δ_H_ 2.11, s and 2.08, s, each 3H). The NMR data of **4** was found to be similar to those of known compound **7** [15], the only difference is the replacement of the hydroxy group at C-3 in **7** by one acetoxy group in **4**. The stereochemistry of compound **4** was also determined by the NOESY spectrum (Figure 3), which exhibited NOE correlations of H-10 with H-1 and H-8β (δ 2.04), H-8β with H_3_-16, establishing the β-orientation of H_3_-16. On the basis of these results and observed NOE correlations (Figure 3), the structure of metabolite **4** was determined.

**Table 2:** ^13^C and ^1^H NMR data for compounds **4** and **5**.

	4		5	
	δ_H_	δ_C_	δ_H_	δ_C_
1	2.18 m	45.7 (CH) ^a^	2.19 m	45.9 (CH)
2	3.63 brs ^b^	92.1 (CH)	3.59 brs	92.3 (CH)
3		86.7 (C)		86.4 (C)
4	2.04 m	35.7 (CH_2_)	1.94 m	35.6 (CH_2_)
	2.60 dd (15.6, 8.8) ^c^		2.63 m	
5	1.26 m	29.2 (CH_2_)	1.37 m	27.4 (CH_2_)
	1.59 m		1.95 m	
6	5.63 d (6.0)	84.7 (CH)	5.22 d (6.0)	83.4 (CH)
7		75.6 (C)		72.9 (C)
8	1.86 m	45.9 (CH_2_)	1.81 dd (14.0, 3.6)	45.2 (CH_2_)
	2.04 m		2.08 m	
9	4.17 q (7.2)	78.1 (CH)	3.85 ddd (3.6, 7.6, 11.2)	78.6 (CH)
10	2.98 t (7.2)	53.8 (CH)	3.06 t (7.6)	53.8 (CH)
11		147.6 (C)		147.4 (C)
12	2.04 m	31.5 (CH_2_)	2.05 m	31.6 (CH_2_)
	2.28 m		2.31 m	
13	1.02 m	24.6 (CH_2_)	1.02 m	24.6 (CH_2_)
	1.72 m		1.72 m	
14	1.29 m	43.9 (CH)	1.23 m	43.9 (CH)
15	1.39 s	22.9 (CH_3_)	1.38 s	23.1 (CH_3_)
16	1.20 s	23.7 (CH_3_)	1.27 s	25.4 (CH_3_)
17	4.62 brs	109.5 (CH_2_)	4.64 brs	109.9 (CH_2_)
	4.68 brs		4.71 brs	
18	1.72 m	29.0 (CH)	1.74 m	29.0 (CH)
19	0.97 d (7.2)	21.9 (CH_3_)	0.98 d (7.2)	21.9 (CH_3_)
20	0.79 d (7.2)	15.4 (CH_3_)	0.79 d (7.2)	15.4 (CH_3_)
3-OAc		169.6 (C)		169.6 (C)
	2.11 s	22.4 (CH_3_)	2.13 s	22.5 (CH_3_)
6-OAc		171.9 (C)		170.1 (C)
	2.08 s	21.4 (CH_3_)	2.08 s	21.3 (CH_3_)

^13^C and ^1^H spectra recorded at 100 and 400 MHz in CDCl_3_; ^a^ Deduced from DEPT; ^b^ Broad signal; ^c^ *J* values (Hz) in parentheses.

The HRESIMS of klymollin X (**5**) exhibited a [M + Na]^+^ ion peak at *m*/*z* 445.2563, which was consistent with the molecular formula of C_24_H_38_O_6_. Furthermore, it was found that the NMR data of **5** (Table 2) were very similar to those of **4**, suggesting that **5** might be a regioisomer of **4**. From NOESY spectrum, it was found that the α-oriented H-9 (δ3.85) showed NOE interaction with H-8α (δ 1.81), and the later exhibited further interaction with H_3_-16. This inferred the α-orientation of the methyl substituent at C-7. Further analysis of other NOE interactions revealed that **5** possessed the same relative configurations at C-1, C-2, C-3, C-9, C-10, C-12 and C-14 as those of **4**. Therefore, compound **5** was found to be the C-7 epimer of **4**.

**Figure 4:**
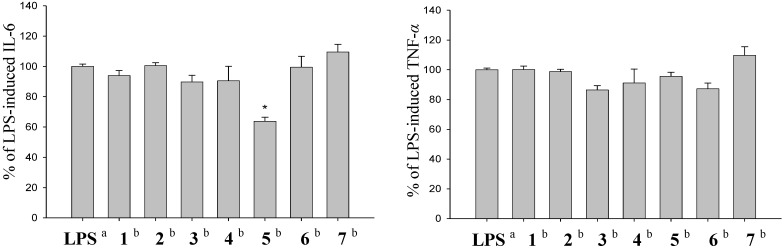
Effect of compounds **1**–**7** on LPS-induced IL-6 and TNF-α expression in RAW264.7 macrophage cells by ELISA analysis. The values are mean ± SEM. (*n* = 6). Relative intensity of the LPS alone stimulated group was taken as 100%. * Significantly different from LPS alone stimulated group (*P* < 0.05). ^a^ stimulated with LPS, ^b^ stimulated with LPS in the presence of **1**–**7** (25 μM).

Eunicellin-type diterpenoids isolated from Formosan soft corals was reported to have anti-inflammatory activities [22]. Therefore, the *in vitro* anti-inflammatory effects of compounds **1**–**7** were tested by examining the inhibitory activity of these compounds toward the LPS-induced up-regulation of pro-inflammatory proteins, IL-6 and TNF-α in murine RAW264.7 macrophage cells (Figure 4). At a concentration of 25 μM, compound **5** significantly reduce the level of IL-6, relative to the control cells stimulated with LPS only. However, these metabolites did not reduce the expression of TNF-α effectively. The cytotoxicity of the diterpenoids **3–7** against five human carcinoma cell lines, CCRF-CEM, K562, Molt 4, T47D and DLD-1 were also evaluated by the MTT assay. Cytotoxicity of **1** and **2** was not measured due to the paucity of these two compounds. Among the tested compounds, **7** showed stronger activity against the proliferation of four cancer cell lines (ED_50_ values of CCRF-CEM, K562, Molt 4 and T47D were 4.2, 15.0, 16.5 and 12.4 μg/mL), and **4** exhibited cytotoxicity toward CCRF-CEM, Molt 4 and T47D cancer cell lines with ED_50_ values of 9.6, 8.5 and 19.9 μg/mL, respectively. These results together with our previous findings [19,20], demonstrated that the soft coral *K. molle* is a good source of bioactive substances which deserve for further biomedical investigations.

## 3. Experimental Section

### 3.1. General Experimental Procedures

Optical rotations were measured on a JASCO P-1020 polarimeter. IR spectra were recorded on a JASCO FT/IR-4100 infrared spectrophotometer. ESIMS and HRESIMS were obtained with a Bruker APEX II mass spectrometer. NMR spectra were recorded in C_6_D_6_ or CDCl_3_, either on a Varian UNITY INOVA-500 FT-NMR, a Varian 400MR FT-NMR, or a Bruker AMX-300 FT-NMR. Silica gel (230–400 mesh, Merck, Darmstadt, Germany) was used for column chromatography. Precoated silica gel plates (Kieselgel 60 F-254, 0.2 mm, Merck, Darmstadt, Germany) were used for analytical TLC (Merck, Darmstadt, Germany). High-performance liquid chromatography (HPLC) was performed on a Hitachi L-2130 HPLC apparatus (Hitachi, Tokyo, Japan) with a Supelco C18 column (250 × 21.2 mm, 5 μm, Supelco, Bellefonte, USA) and a Hitachi L-2455 diode array detector (Hitachi, Tokyo, Japan).

### 3.2. Animal Material

The soft coral *Klyxum molle* was collected by hand using scuba along the coast of Peng-Hu Islands, Taiwan, in June 2008 at a depth of 10 m, and was stored in a freezer until extraction. A voucher sample (PI-20080610) was deposited at the Department of Marine Biotechnology and Resources, National Sun Yat-sen University.

### 3.3. Extraction and Separation

The frozen bodies of *K. molle* (1.3 kg, wet weight) were sliced and exhaustively extracted with EtOAc (3 × 10 L). The organic extract was concentrated to an aqueous suspension and was partitioned between EtOAc and H_2_O. The EtOAc layer was dried with anhydrous Na_2_SO_4_. After removal of solvent *in vacuo*, the residue (22 g) was subjected to column chromatography on silica gel and eluted with EtOAc in *n*-hexane (0%–100% of EtOAc, gradient) and further with MeOH in EtOAc of increasing polarity to yield 31 fractions. Fraction 18, eluted with *n*-hexane–EtOAc (8:1), was further chromatographed over silica gel with a gradient elution using a minture of *n*-hexane–acetone (7:1) to afford three subfractions (F18B1–F18B3) and compound **7** (39.6 mg). Subfractions F18B1 was subjected to reversed-phase HPLC (CH_3_CN–H_2_O, 1.2:1 to 1.6:1) in order to purify compounds **3** (4.0 mg), **4** (7.4 mg), **5** (4.7 mg) and **6** (9.2 mg). Fraction 20, obtained from *n*-hexane–EtOAc (1:2), was further purified over silica gel using *n*-hexane–acetone (4:1) to afford four subfractions (F20B1–F20B4). Subfraction F20B4 was separated by reversed-phase HPLC (CH_3_CN–H_2_O, 1:1) to afford compounds **1** (1.5 mg) and **2** (1.7 mg).

Klymollin T (**1**): colorless oil; 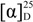 −67 (*c* 0.15, CHCl_3_); IR (neat) ν_max_ 3481, 2956, 2922, 2874, 2852, 1746, 1456, 1372, 1240, 1098 and 1043 cm^−1^; ^13^C and ^1^H NMR data (500 MHz; C_6_D_6_), see Table 1; ESIMS *m/z* 545 [M + Na]^+^; HRESIMS *m/z* 545.2722 [M + Na]^+^ (calcd for C_27_H_40_O_11_Na, 525.2726).

Klymollin U (**2**): colorless oil; 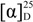 −57 (*c* 0.17, CHCl_3_); IR (neat) ν_max_ 3481, 2958, 2927, 2875, 2854, 1735, 1456, 1370, 1243, 1098 and 1043 cm^−1^; ^13^C and ^1^H NMR data (400 MHz; CDCl_3_), see Table 1; ESIMS *m/z* 545 [M + Na]^+^; HRESIMS *m/z* 545.2723 [M + Na]^+^ (calcd for C_27_H_40_O_11_Na, 545.2726).

Klymollin V (**3**): colorless oil; 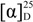 −18 (*c* 1.14, CHCl_3_); IR (neat) ν_max_ 2953, 2931, 2877, 1735, 1636, 1467, 1373, 1227, 1183, 1082 and 1038 cm^–1^; ^13^C and ^1^H NMR data (400 MHz; CDCl_3_), see Table 1; LRESIMS *m/z* 357 [M + Na]^+^; HRESIMS *m/z* 357.2405 [M + Na]^+^ (calcd for C_21_H_34_O_3_Na, 357.2406).

Klymollin W (**4**): colorless oil; 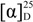 +14 (*c* 2.11, CHCl_3_); IR (neat) ν_max_ 3466, 2959, 2935, 2872, 1732, 1644, 1448, 1370, 1250, 1103, 1049 and 1023 cm^–1^; ^13^C and ^1^H NMR data (400 MHz; CDCl_3_), see Table 2; LRESIMS *m/z* 445 [M + Na]^+^; HRESIMS *m/z* 445.2563 [M + Na]^+^ (calcd for C_24_H_38_O_6_Na, 445.2566).

Klymollin X (**5**): colorless oil; 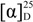 +18 (*c* 1.34, CHCl_3_); IR (neat) ν_max_ 3478, 2959, 2932, 2871, 1735, 1645, 1431, 1371, 1115 and 1021 cm^–1^; ^13^C and ^1^H NMR data (400 MHz; CDCl_3_), see Table 2; LRESIMS *m/z* 445 [M + Na]^+^; HRESIMS *m/z* 445.2568 [M + Na]^+^ (calcd for C_24_H_38_O_6_Na, 445.2566).

### 3.4. Cytotoxicity Testing

Cell lines were purchased from the American Type Culture Collection (ATCC). Cytotoxicity assays of **3**–**7** were performed using the MTT [3-(4,5-dimethylthiazol-2-yl)-2,5 diphenyltetrazolium bromide] colorimetric method [23,24].

### 3.5. In Vitro Anti-Inflammatory Assay

Mouse macrophage cell line, RAW264.7, was purchased from ATCC. *In vitro* anti-inflammatory activities of compounds **1**–**7** were measured by examining the inhibition of LPS induced upregulation of IL-6 and TNF-α in macrophages cells [25].

## 4. Conclusions

New eunicellin-based diterpenoids were isolated together with known compounds from the soft coral *Klyxum molle*. Compound **5** could significantly inhibit the release of IL-6 in LPS-induced mouse RAW264.7 macrophage cell line. Also, compounds **4** and **7** showed moderate to weak cytotoxicity.

## Supplementary Files

Supplementary File 1 Supplementary Information (PDF, 1357 KB)
