# Supplementary material for: Klymollins T–X, Bioactive Eunicellin-Based Diterpenoids from the Soft Coral Klyxum molle
Source: Mar Drugs. 2014 May 22;12(5):3060–71. doi: 10.3390/md12053060 (PMC4052331; doi:10.3390/md12053060)
Supplement: Supplementary File 1 — Supplementary Information (PDF, 1357 KB) [file marinedrugs-12-03060-s001.pdf]

Supplementary Information

Figure S1. ^{1}H NMR spectrum of **1** in C_6D_6 at 500 MHz.

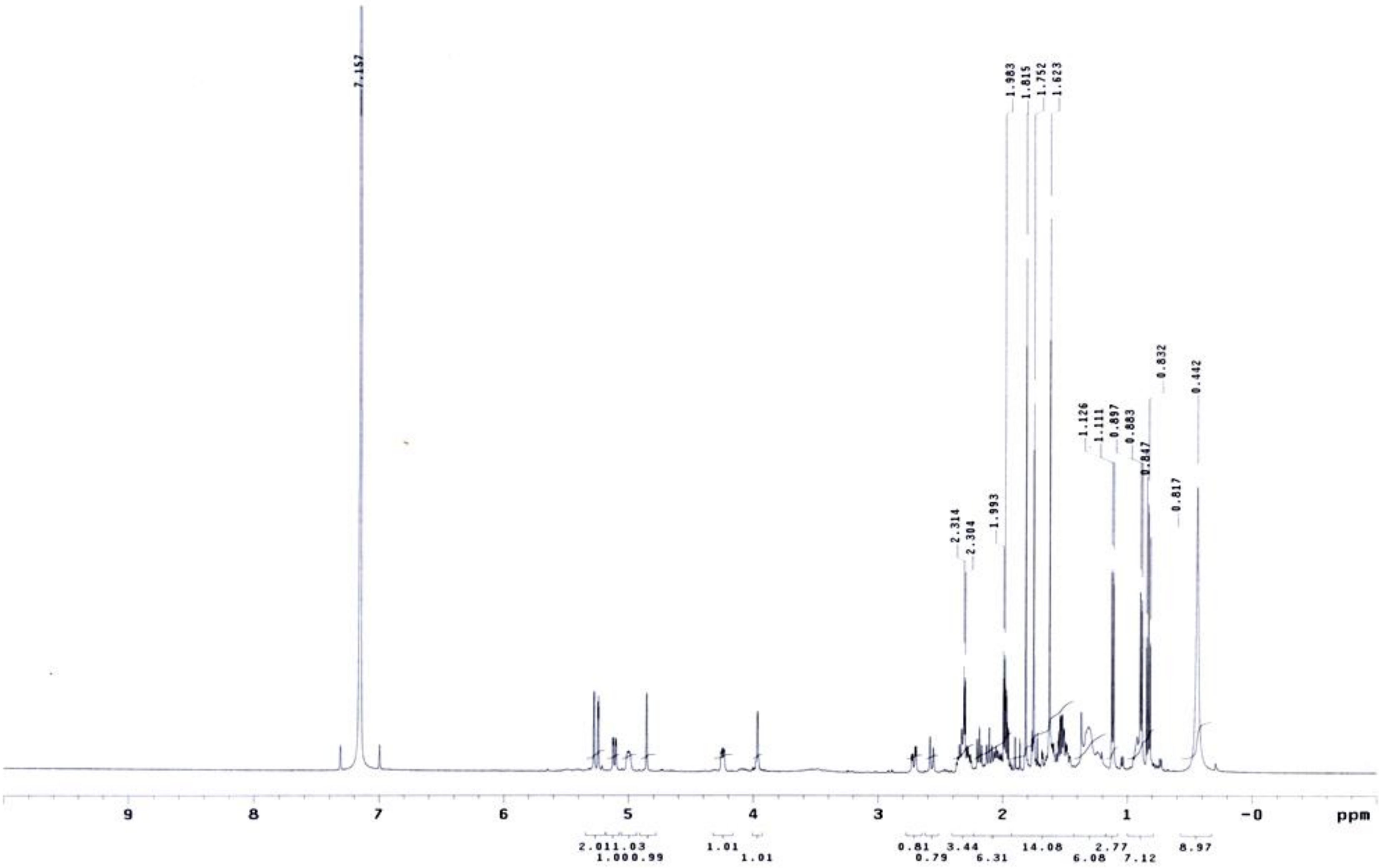

Figure S2. ^{13}C NMR spectrum of **1** in C_6D_6 at 125 MHz.

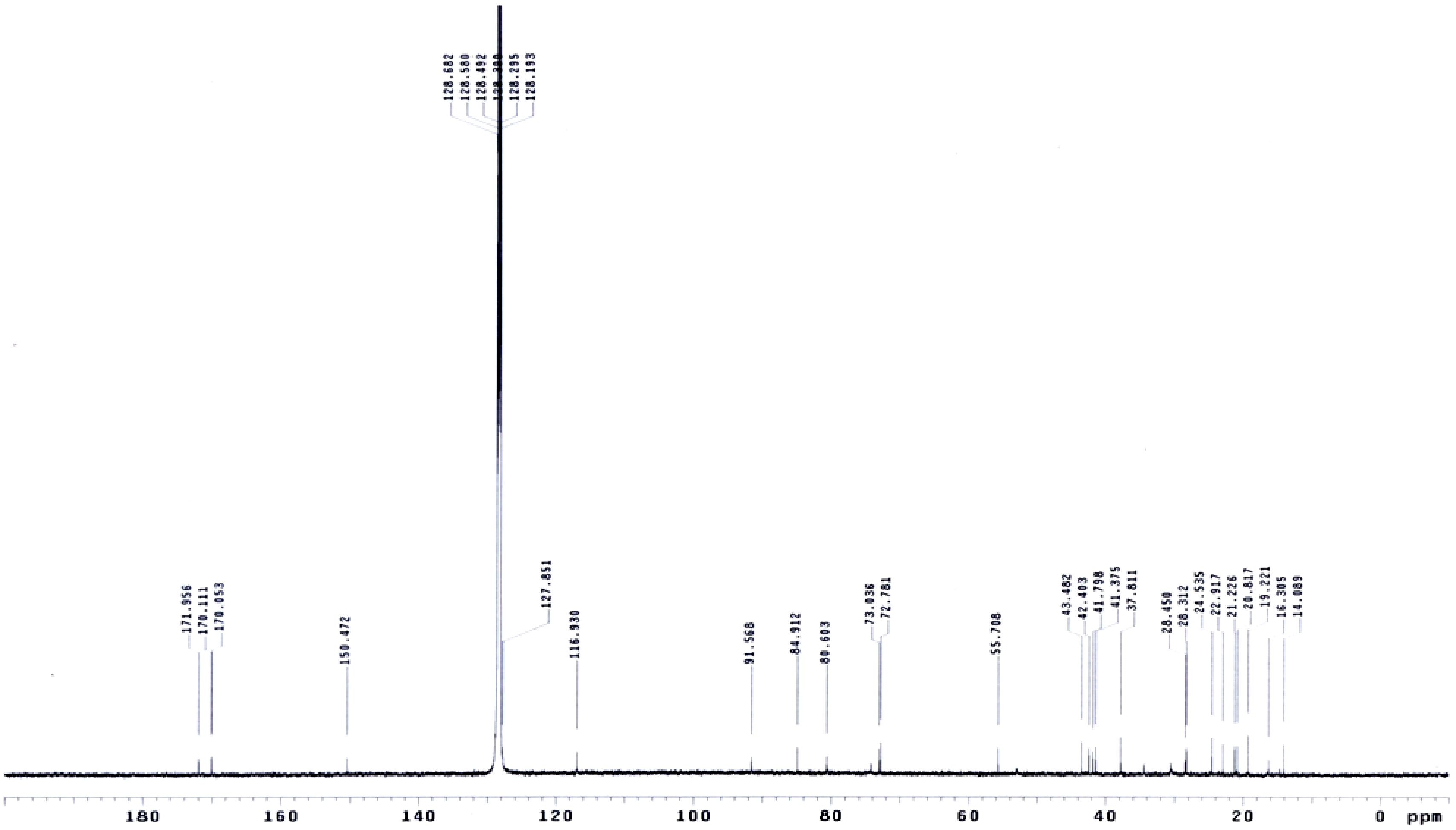

Figure S3. HRESIMS spectrum of **1**.

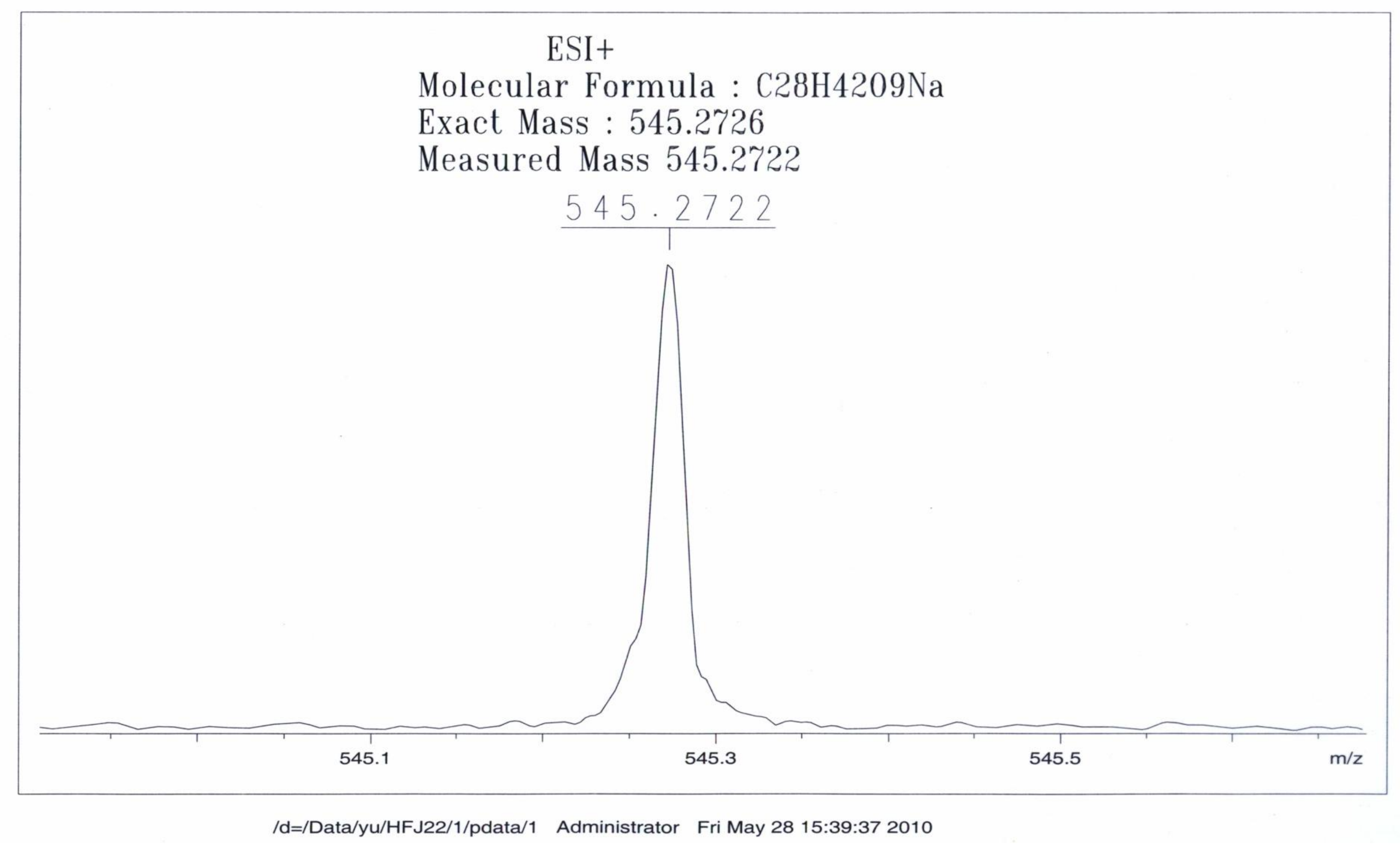

Figure S4. ^{1}H NMR spectrum of **2** in $CDCl_3$ at 400 MHz.

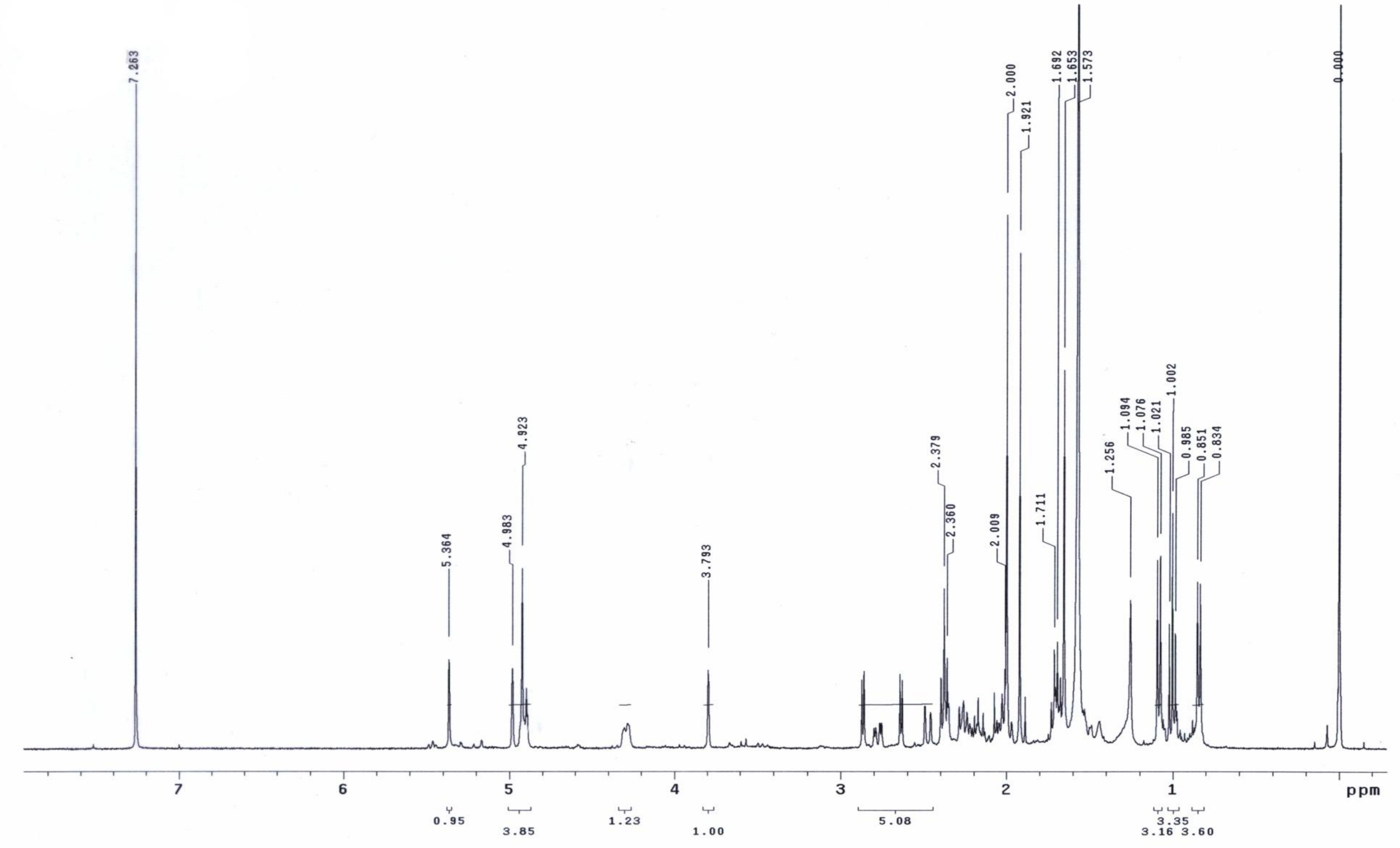

Figure S5. ^{13}C NMR spectrum of **2** in $CDCl_3$ at 100 MHz.

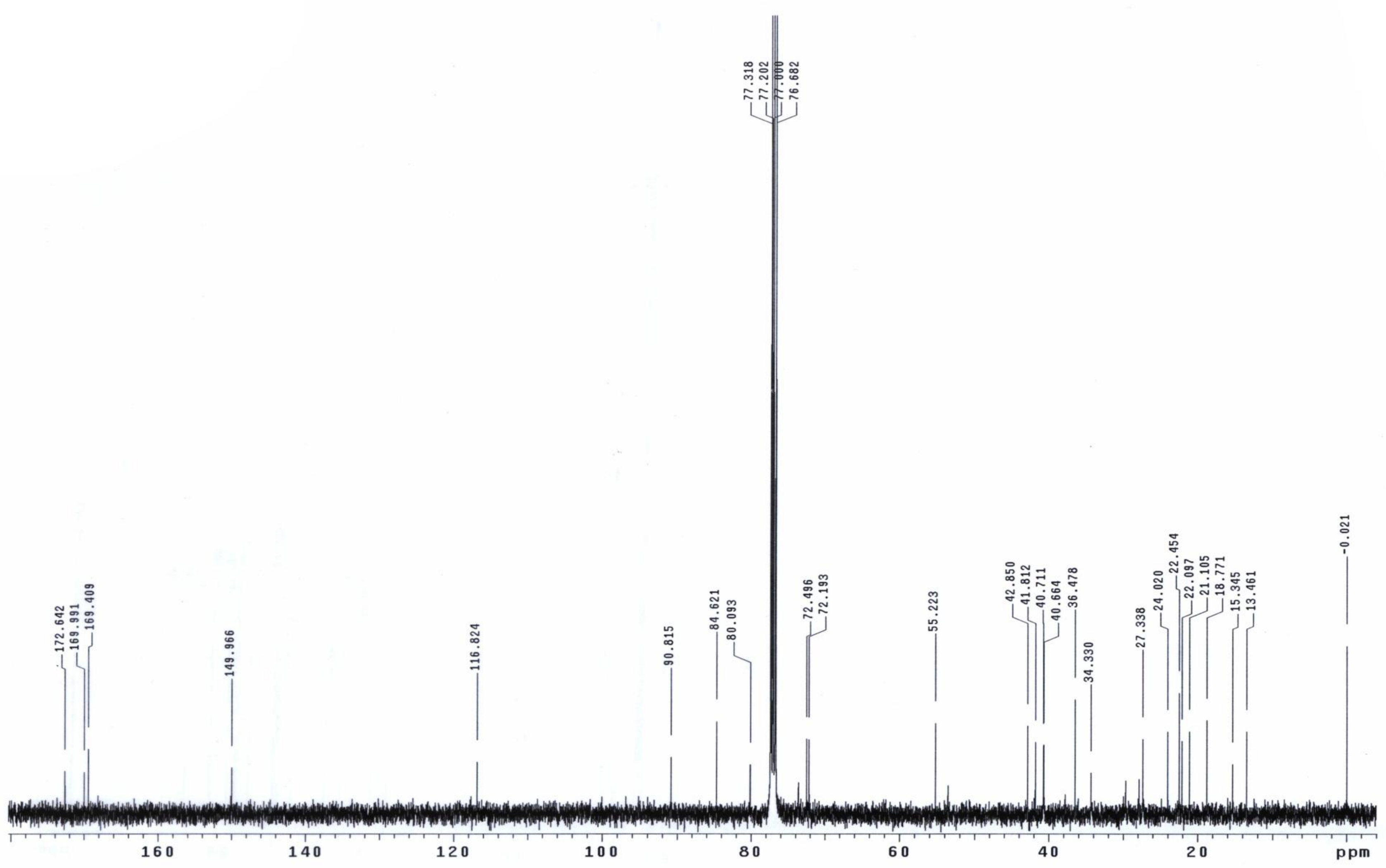

Figure S6. HRESIMS spectrum of **2**.

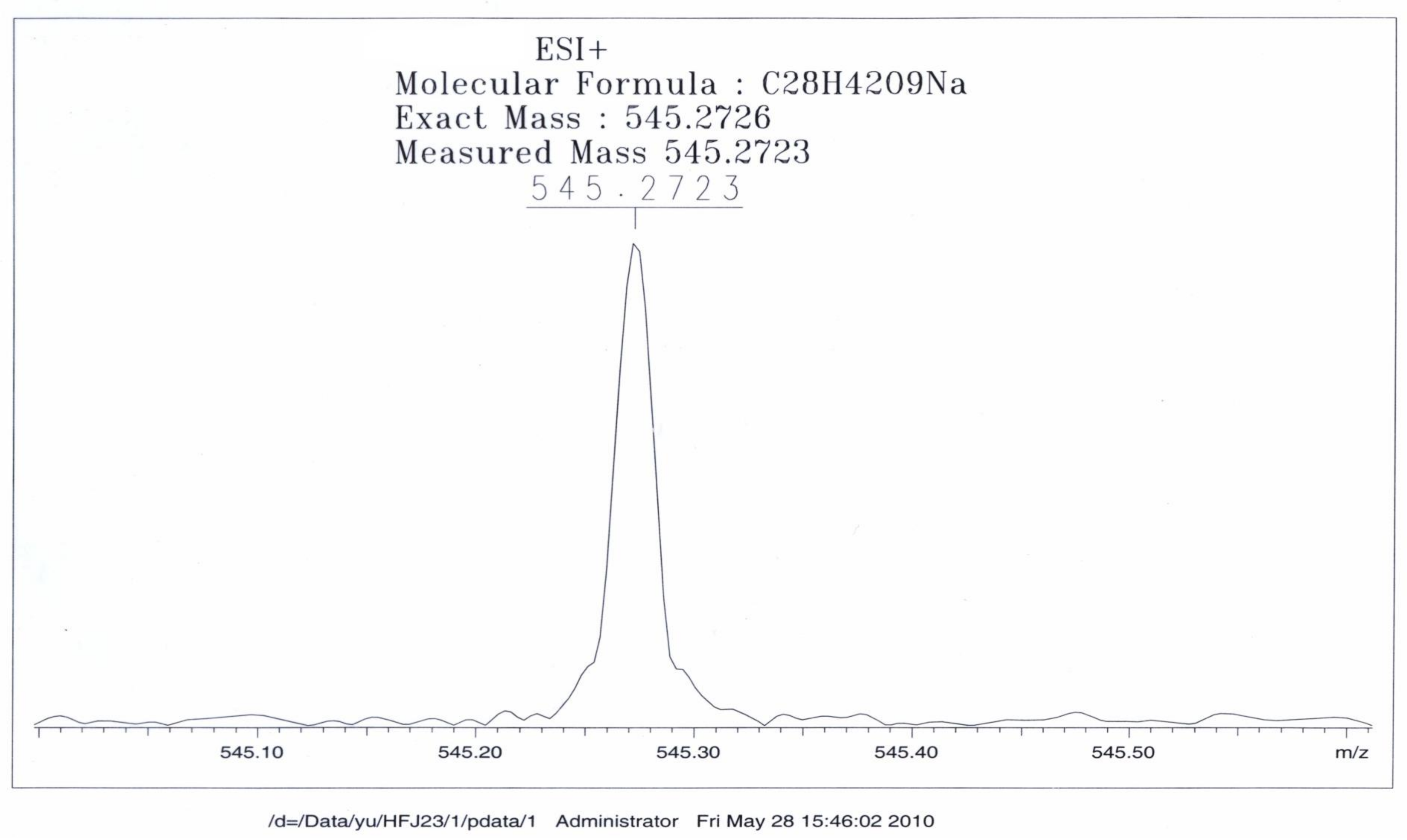

Figure S7. ^{1}H NMR spectrum of **3** in $CDCl_3$ at 400 MHz.

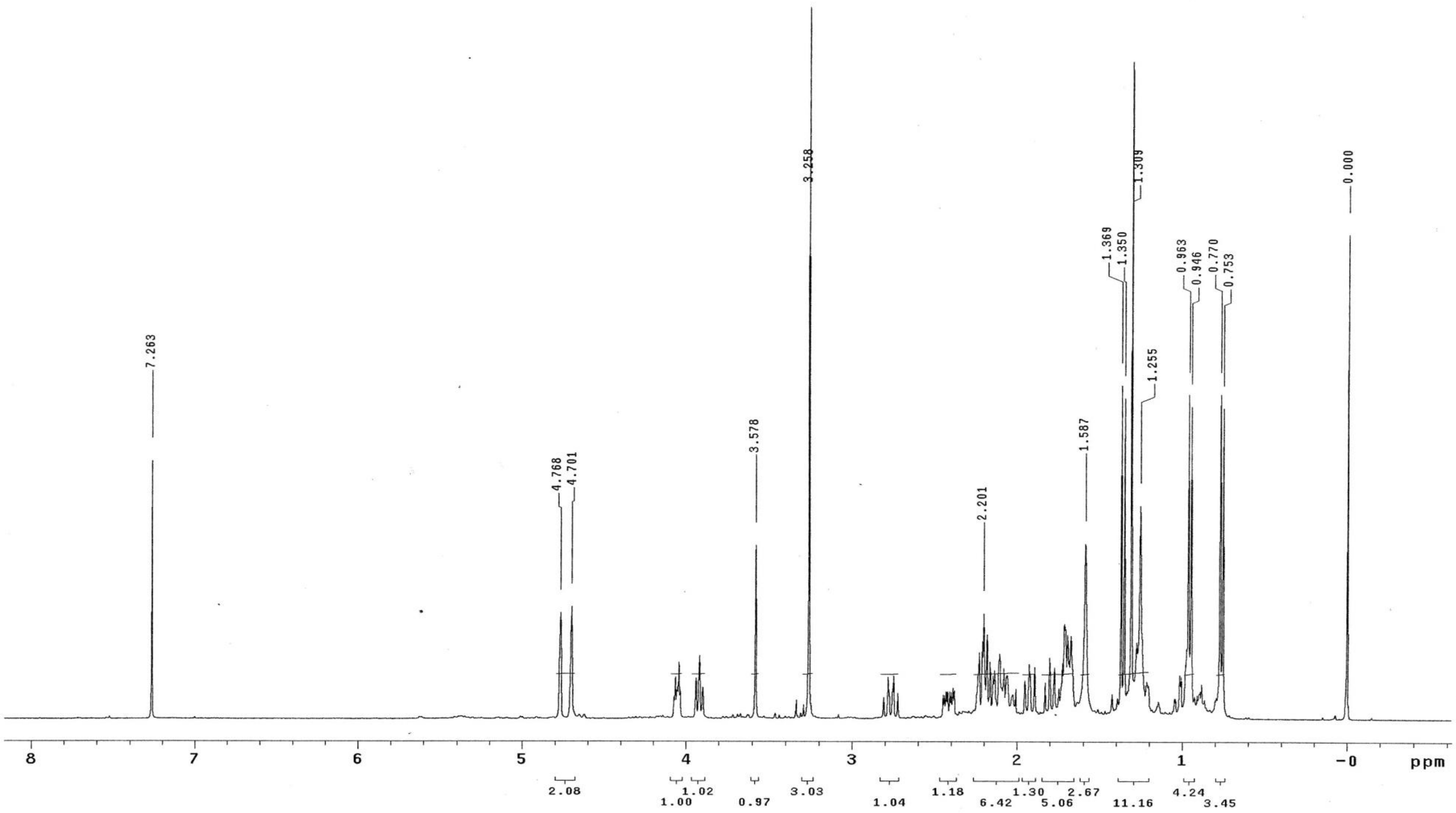

Figure S8. ^{13}C NMR spectrum of **3** in $CDCl_3$ at 100 MHz.

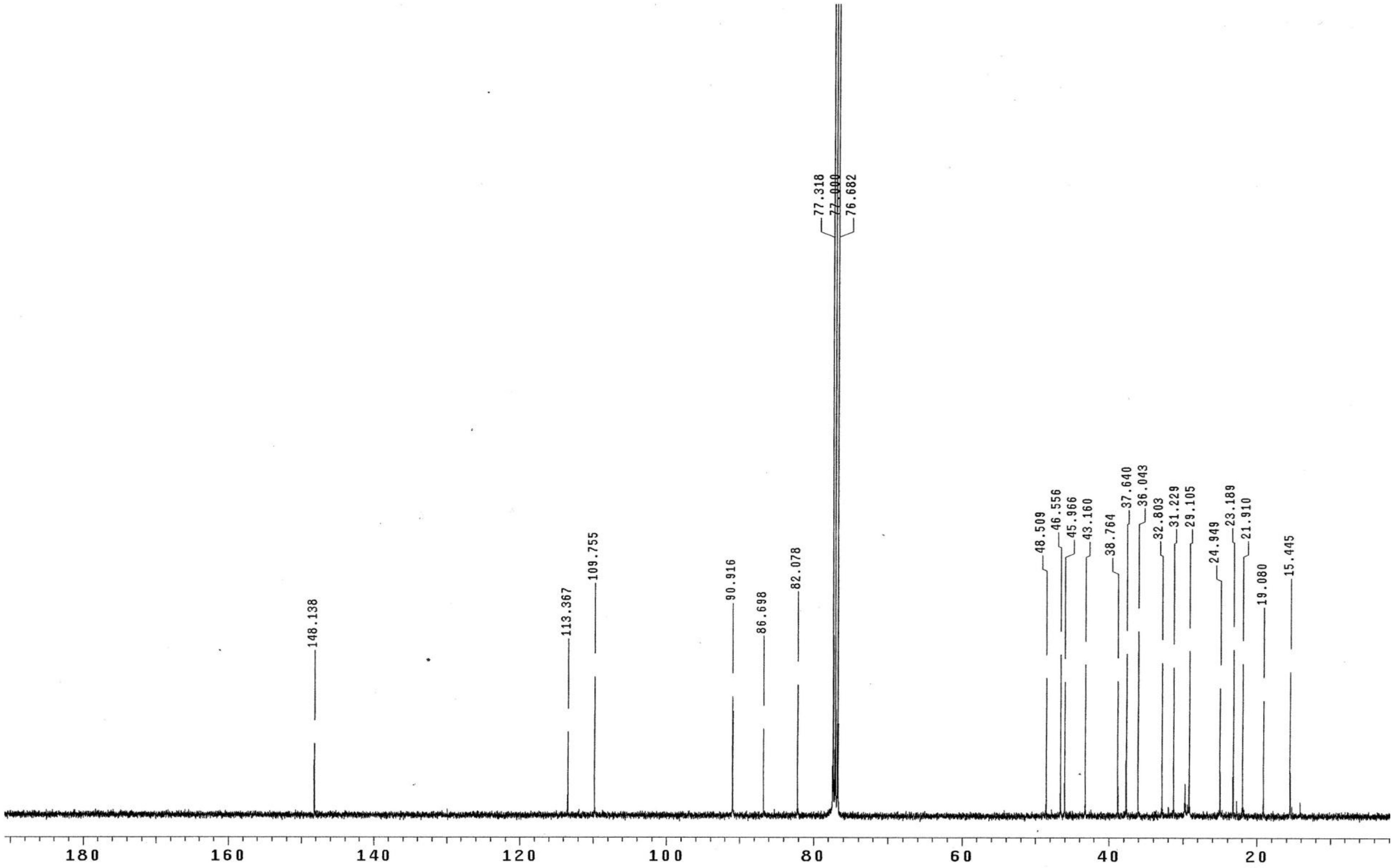

Figure S9. HRESIMS spectrum of **3**.

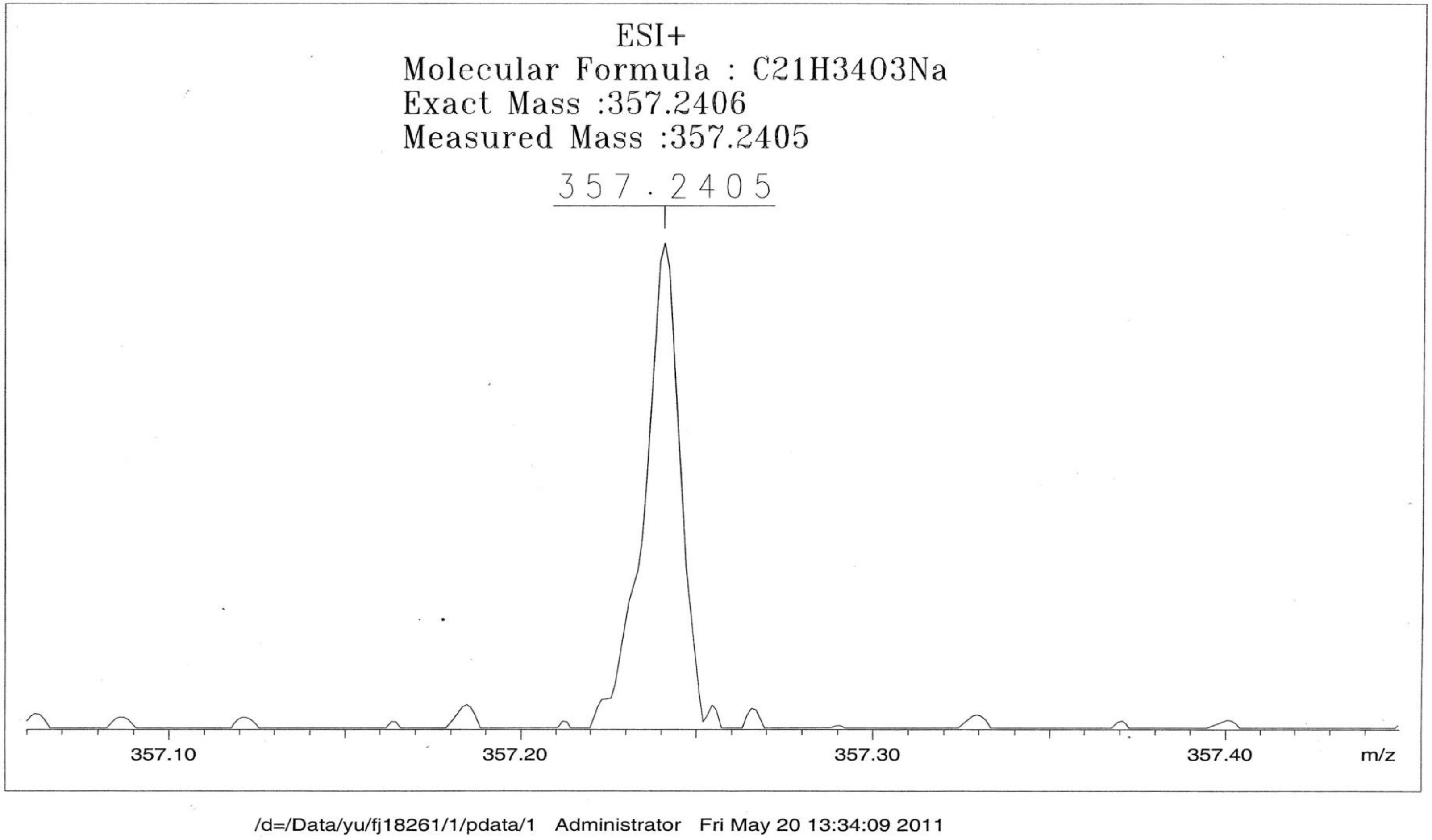

Figure S10. ^{1}H NMR spectrum of **4** in $CDCl_3$ at 400 MHz.

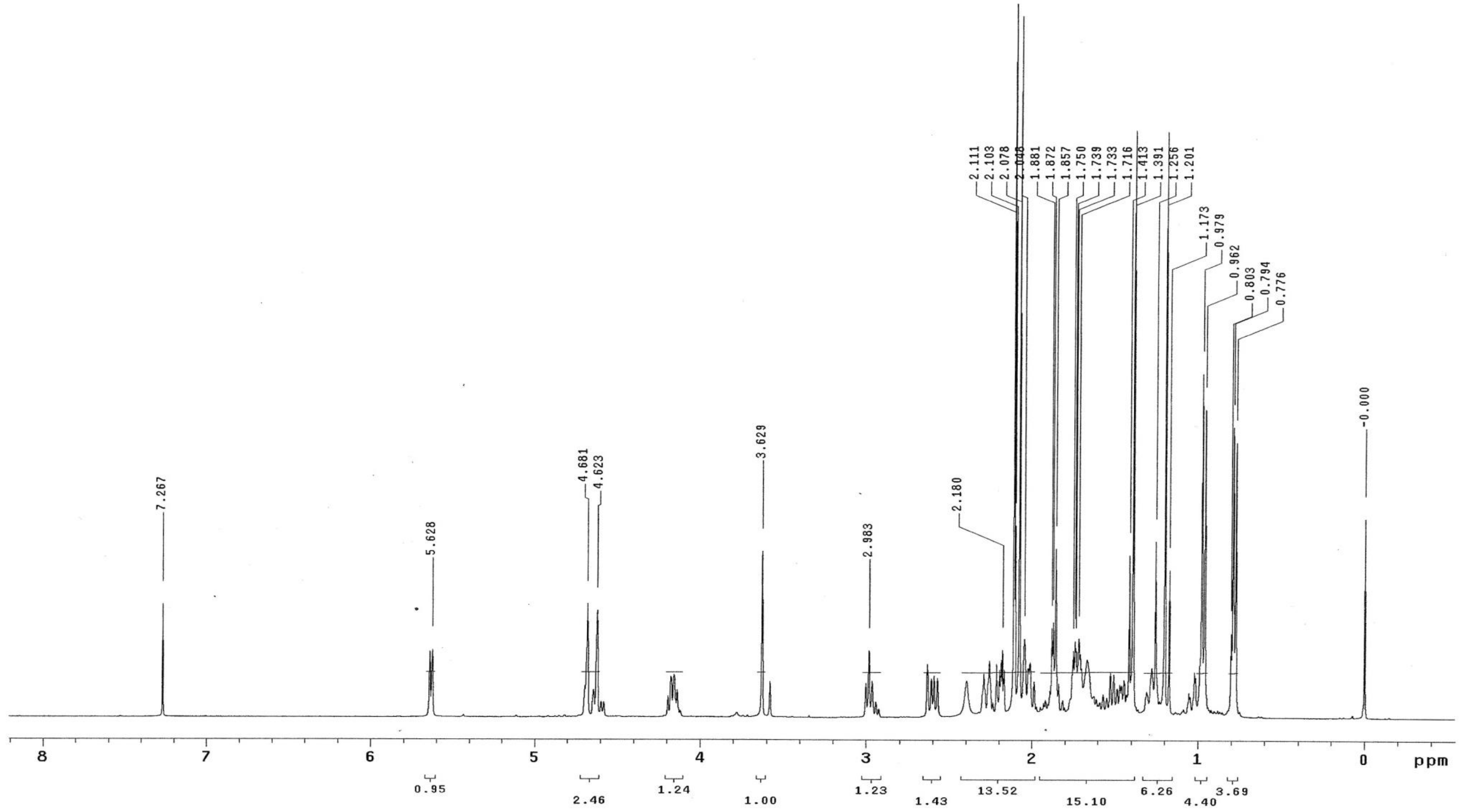

Figure S11. ^{13}C NMR spectrum of **4** in $CDCl_3$ at 100 MHz.

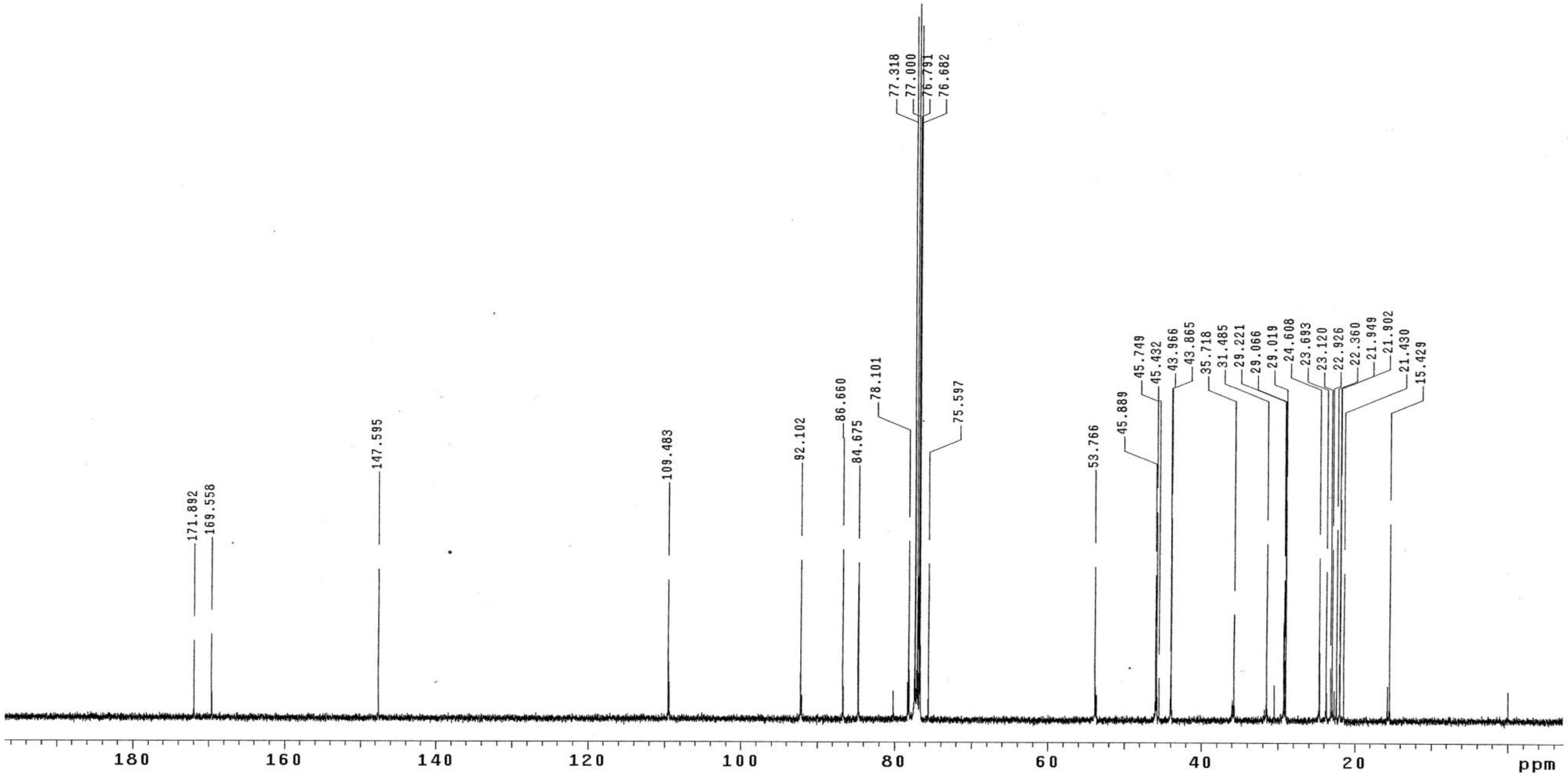

Figure S12. HRESIMS spectrum of **4**.

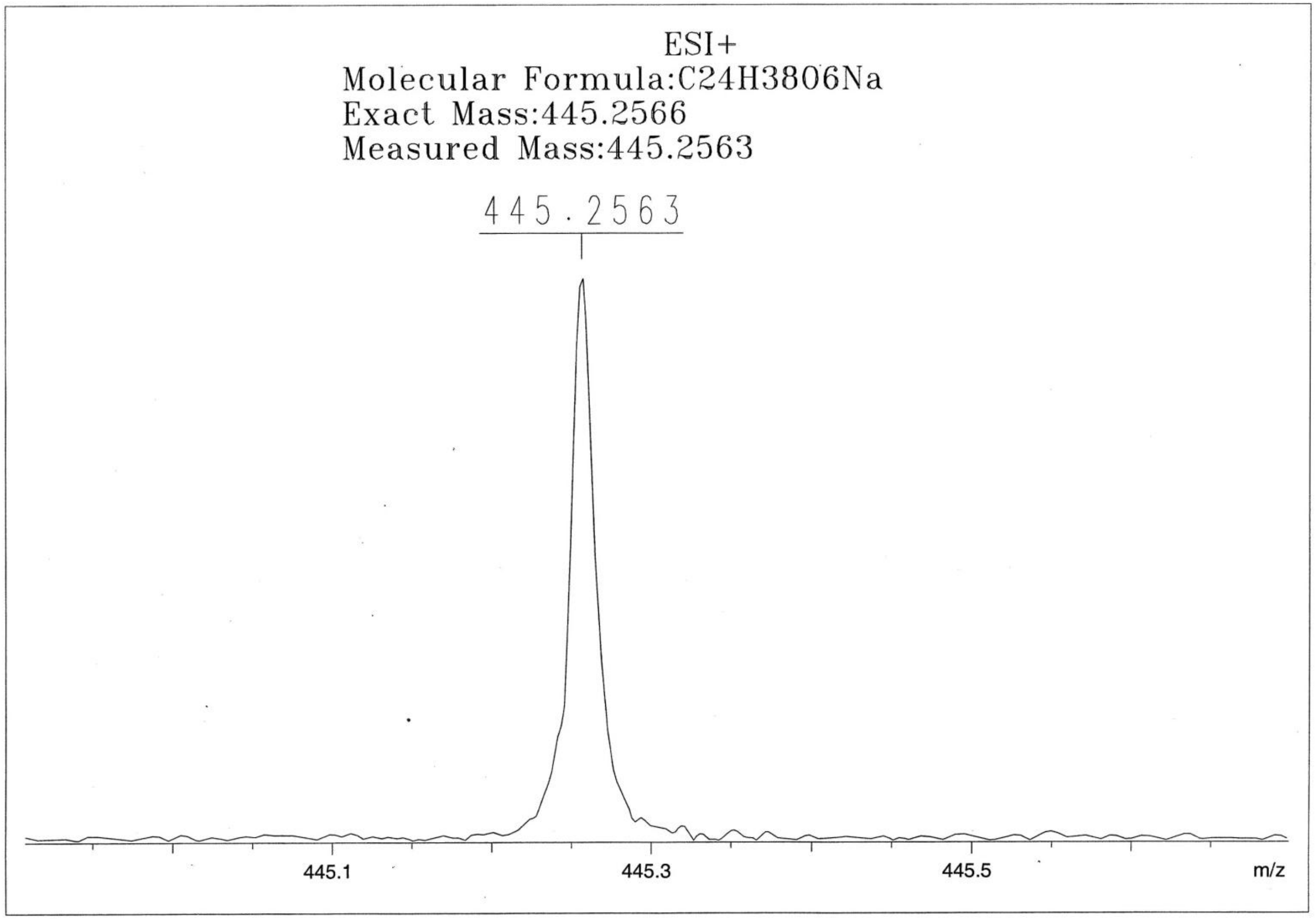

/d=/Data/yu/fj18265962/1/pdata/1 Administrator Wed Jul 13 16:48:10 2011

Figure S13. ^{1}H NMR spectrum of **5** in $CDCl_3$ at 400 MHz.

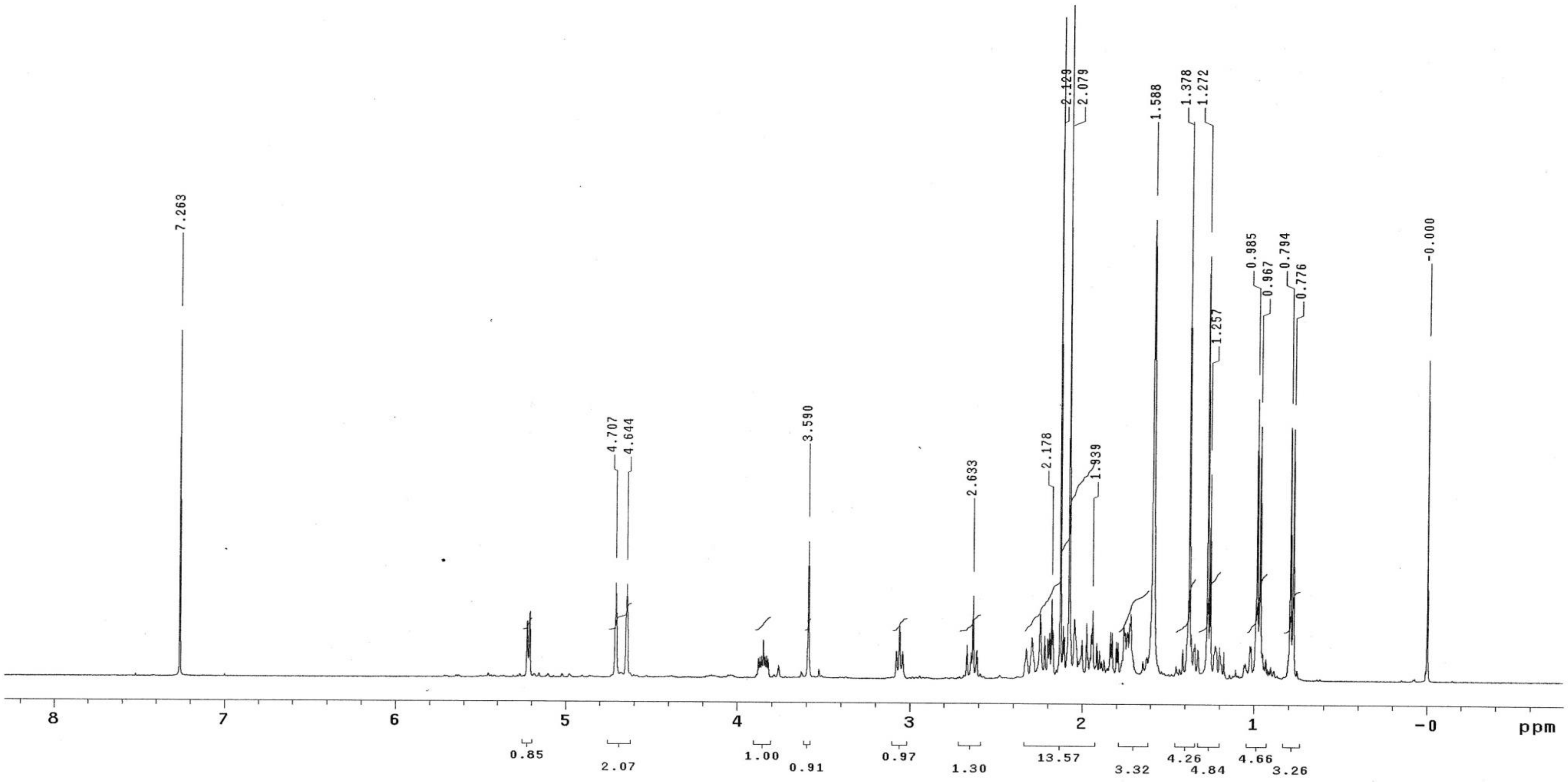

Figure S14. ^{13}C NMR spectrum of **5** in $CDCl_3$ at 100 MHz.

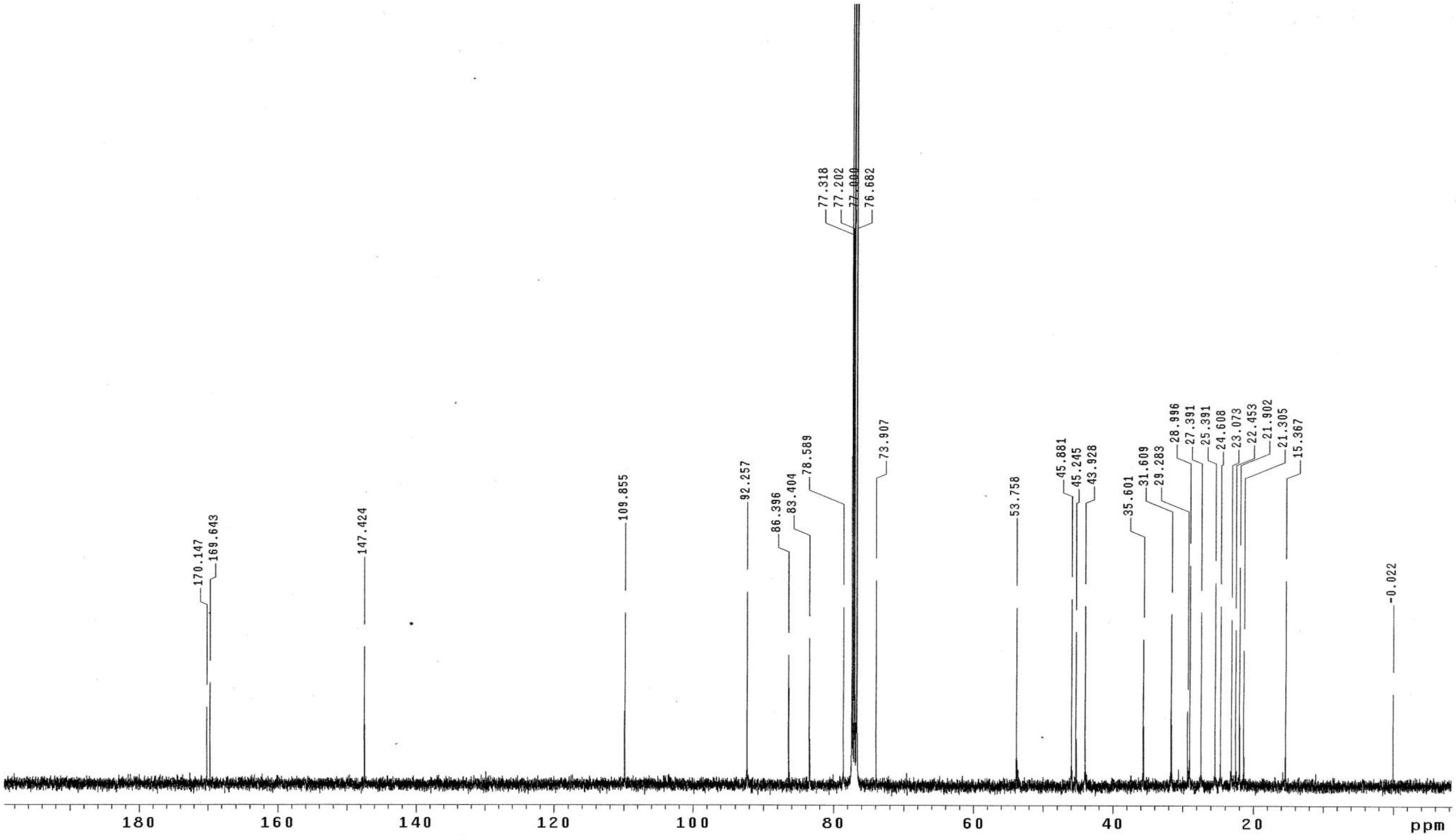

Figure S15. HRESIMS spectrum of **5**.

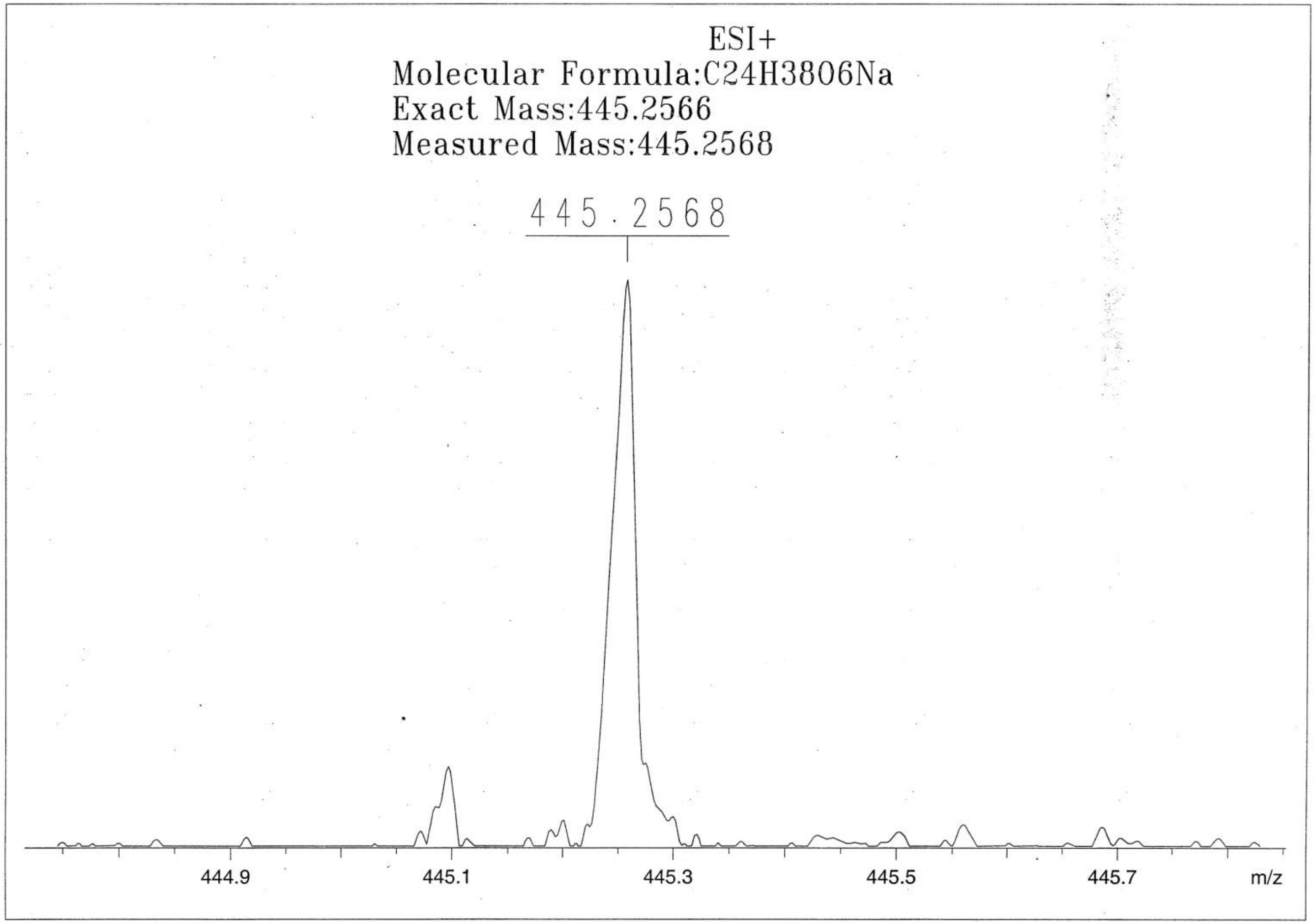

/d=/Data/yu/FJ182671091/1/pdata/1 Administrator Fri Feb 10 14:02:57 2012

© 2014 by the authors; licensee MDPI, Basel, Switzerland. This article is an open access article distributed under the terms and conditions of the Creative Commons Attribution license (http://creativecommons.org/licenses/by/3.0/).
